# Supplementary material for: The Relationship between Low-Sodium Salt Intake and Both Blood Pressure Level and Hypertension in Chinese Residents
Source: Nutrients. 2024 Jun 17;16(12):1909. doi: 10.3390/nu16121909 (PMC11206867; doi:10.3390/nu16121909)
Supplement: Supplementary file 1 [file nutrients-16-01909-s001.zip › nutrients-3052990-supplementary.pdf]

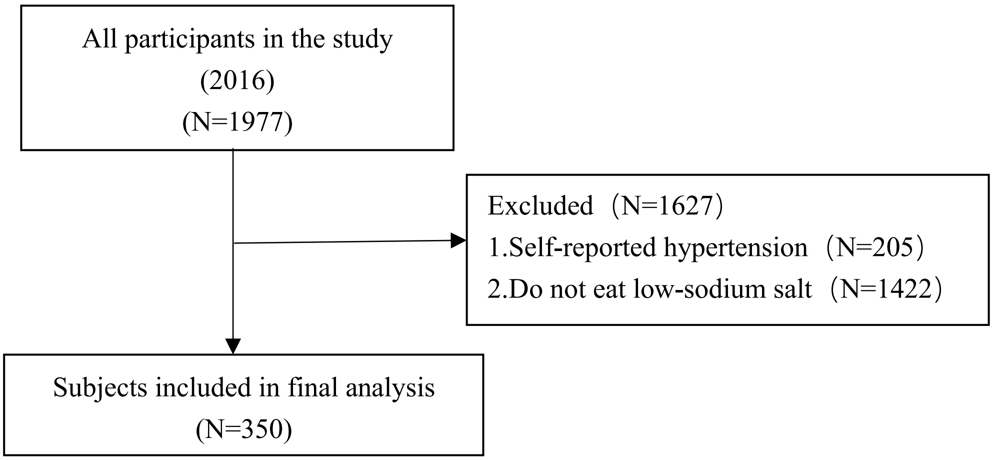

Figure S1 Flowchart of the study population

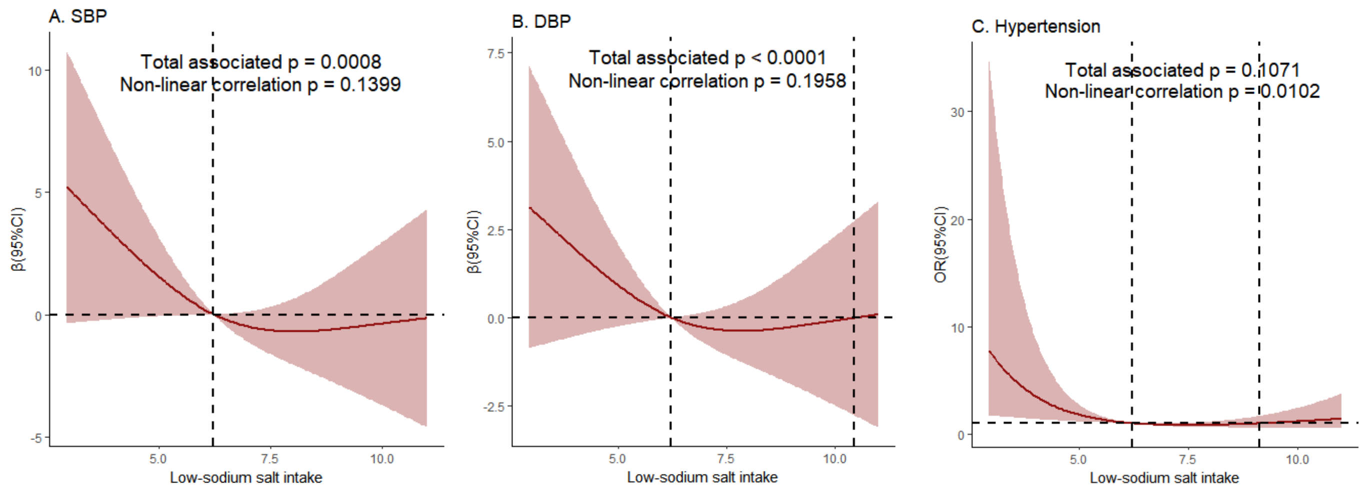

Figure S2 Restricted cubic spline results for males. A: Relationship between low-sodium salt intake and SBP; B: The relationship between low-sodium salt intake and DBP; C: The relationship between low-sodium salt intake and the risk of hypertension. The model was adjusted for age, region, marital status, education level, income, smoking status, drinking status, physical exercise, BMI, dyslipidemia, diabetes and central obesity.

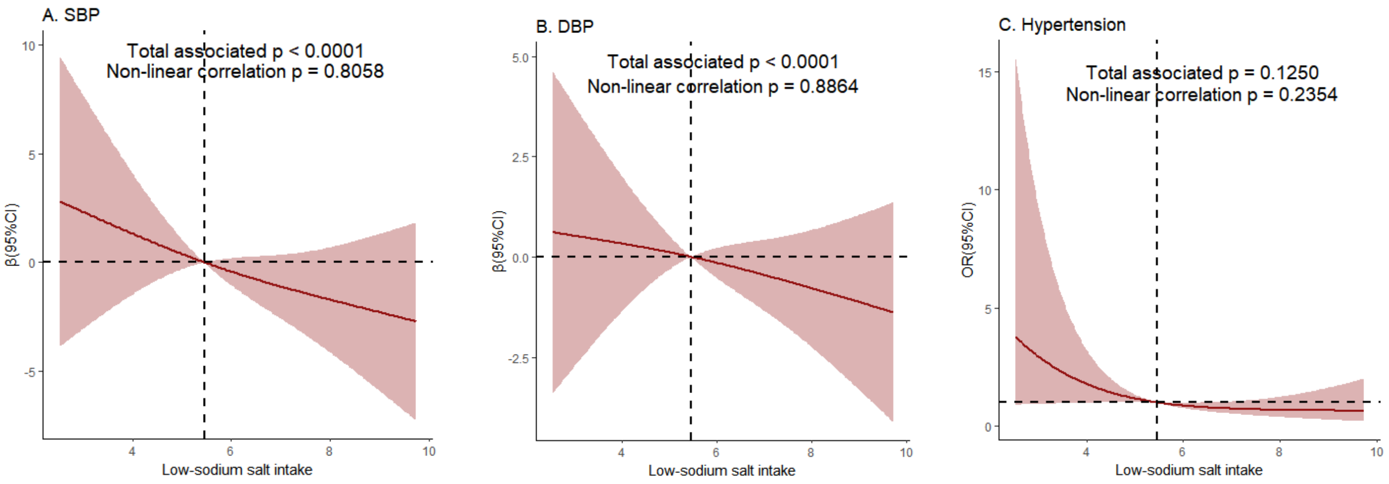

Figure S3 Restricted cubic spline results for females. A: Relationship between low-sodium salt intake and SBP; B: The relationship between low-sodium salt intake and DBP; C: The relationship between low-sodium salt intake and the risk of hypertension. The model was adjusted for age, region, marital status, education level, income, smoking status, drinking status, physical exercise, BMI, dyslipidemia, diabetes and central obesity.

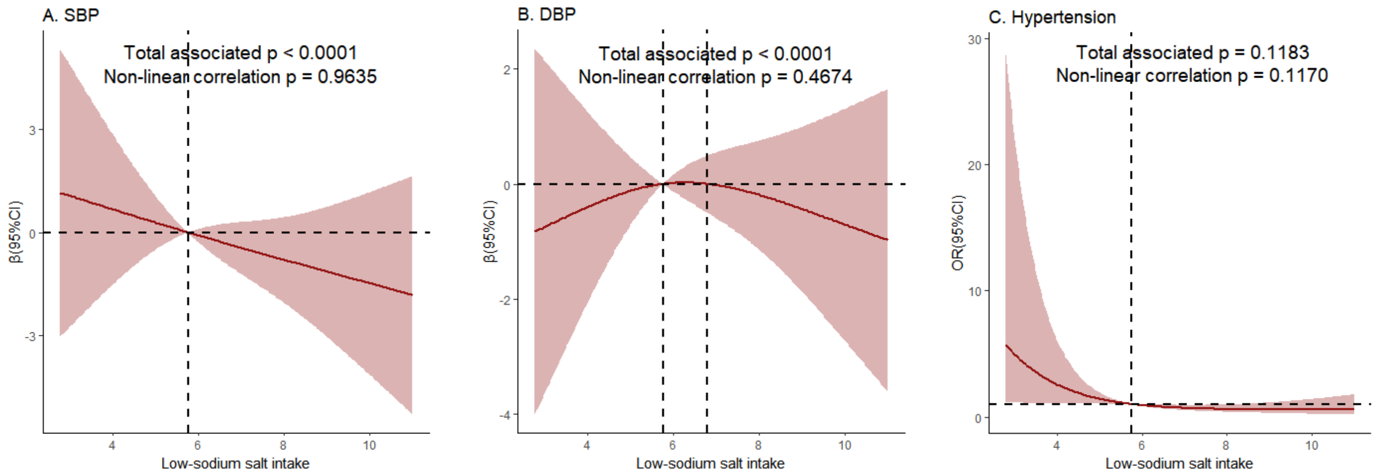

Figure S4 Restricted cubic spline results for people aged 17-44 years. A: Relationship between low-sodium salt intake and SBP; B: The relationship between low-sodium salt intake and DBP; C: The relationship between low-sodium salt intake and the risk of hypertension. The model was adjusted for gender, region, marital status, education level, income, smoking status, drinking status, physical exercise, BMI, dyslipidemia, diabetes and central obesity.

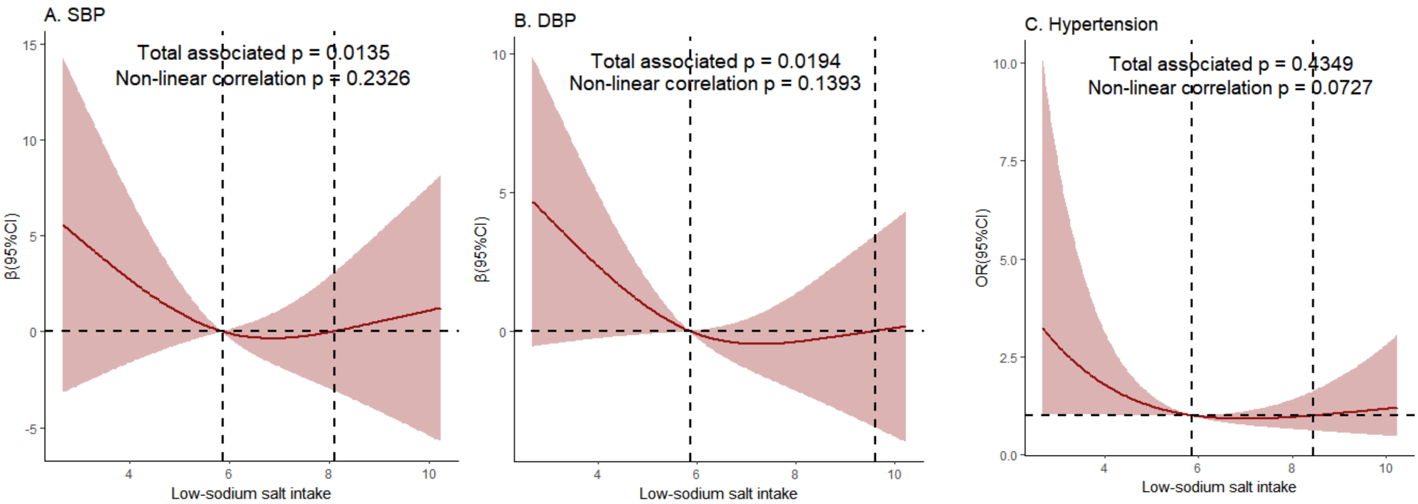

Figure S5 Restricted cubic spline results for people aged 45-70 years. A: Relationship between low-sodium salt intake and SBP; B: The relationship between low-sodium salt intake and DBP; C: The relationship between low-sodium salt intake and the risk of hypertension. The model was adjusted for gender, region, marital status, education level, income, smoking status, drinking status, physical exercise, BMI, dyslipidemia, diabetes and central obesity.

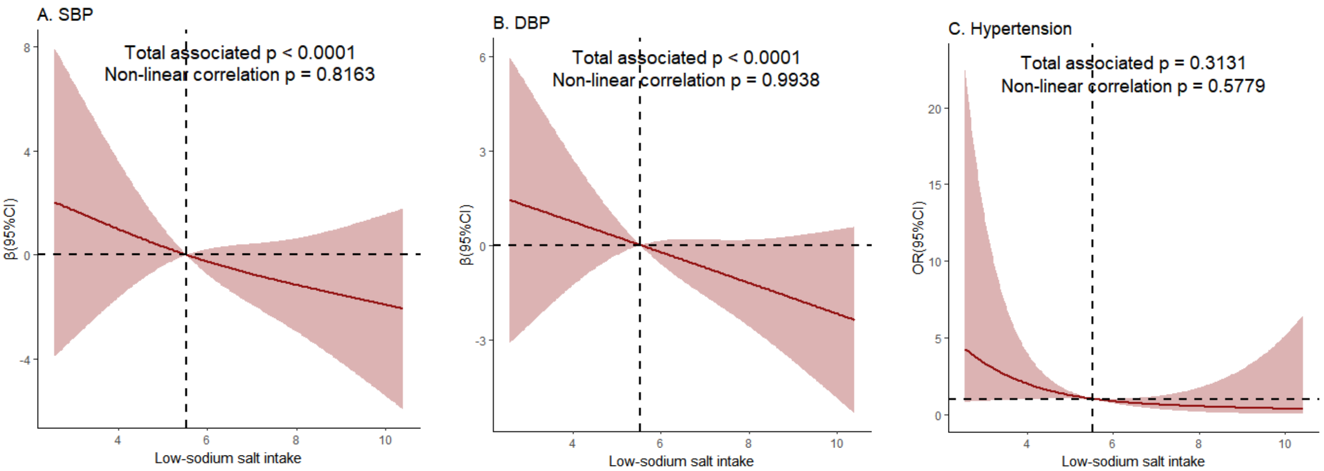

Figure S6 Restricted cubic spline results for people in the urban. A: Relationship between low-sodium salt intake and SBP; B: The relationship between low-sodium salt intake and DBP; C: The relationship between low-sodium salt intake and the risk of hypertension. The model was adjusted for gender, age, marital status, education level, income, smoking status, drinking status, physical exercise, BMI, dyslipidemia, diabetes and central obesity.

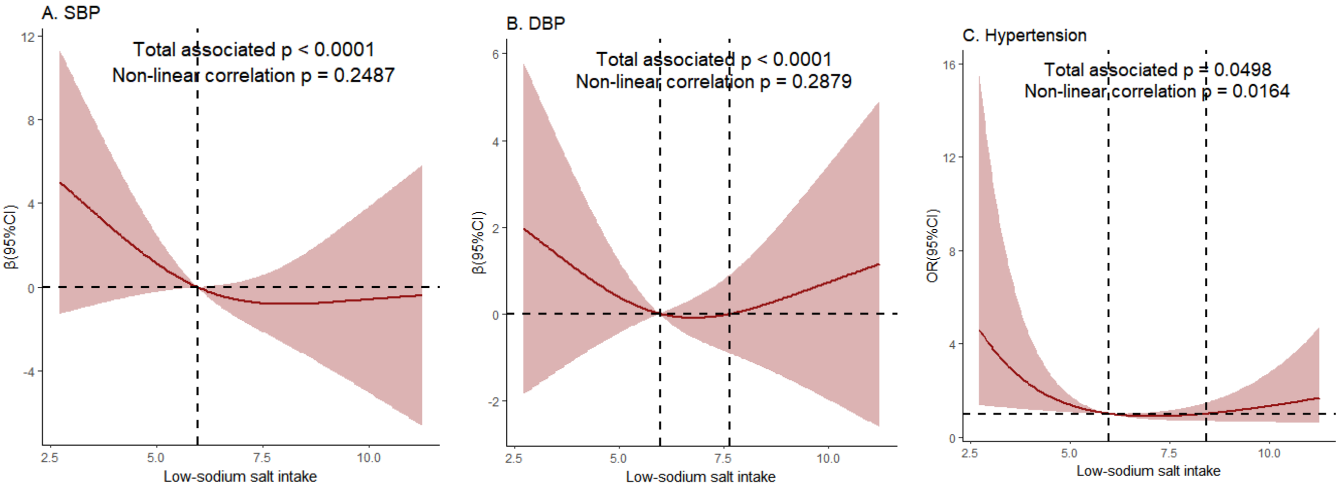

Figure S7 Restricted cubic spline results for people in the rural. A: Relationship between low-sodium salt intake and SBP; B: The relationship between low-sodium salt intake and DBP; C: The relationship between low-sodium salt intake and the risk of hypertension. The model was adjusted for gender, age, marital status, education level, income, smoking status, drinking status, physical exercise, BMI, dyslipidemia, diabetes and central obesity.

Table S1 Variable definition

Variable	Code	Definition
Low-sodium salt intake	1	< 4.72 g/d
	2	≥ 4.72 g/d， < 6.88 g/d
	3	≥ 6.88 g/d
Age groups	1	17-44 years old
	2	45-70 years old
Gender	0	Female
	1	Male
Region	1	Urban
	2	Rural
Marital status	1	Unmarried (single, divorced, separated, or widowed)
	2	Married
Educational level	1	Primary and below
	2	Junior high school
	3	High school and above
Income	1	≥ ¥0, ≤ ¥5000
	2	> ¥5000, ≤ ¥10000
	3	> ¥10000, ≤ ¥15000
	4	> ¥15000
BMI groups	1	< 24 kg/m^2
	2	≥ 24 kg/m^2, < 28 kg/m^2
	3	≥ 28 kg/m^2
Smoking status	0	No (current non-smoker)
	1	Yes (current smoker)
Drinking status	0	No (current non- drinker)
	1	Yes (current drinker)
Physical exercise	0	No (infrequent exercise)
	1	Yes (current exerciser)
Dyslipidemia	0	No
	1	Yes
Diabetes	0	No
	1	Yes
Central obesity	0	No
	1	Yes
Hypertension	0	No
	1	Yes

Table S2 Characteristics of 1772 participants according to low-sodium salt intake

Characteristics	Total cases	Those who do not consume low-sodium salt	Those who consume low-sodium salt	*p-Value* [1]
number of participants, *N*	1772	1422	350	
salt intake (mean (SD))	10.21 (4.95)	10.59 (5.04)	8.67 (4.24)	***<0.001***
low-sodium salt intake (mean (SD))	1.22 (2.77)	0.00 (0.00)	6.16 (2.92)	***<0.001***
SBP (mean (SD))	119.41 (16.49)	119.32 (16.17)	119.76 (17.77)	*0.653*
DBP (mean (SD))	76.24 (11.20)	76.20 (11.06)	76.43 (11.76)	*0.727*
hypertension, N%				
no	1509 (85.2)	1207 (84.9)	302 (86.3)	*0.563*
yes	263 (14.8)	215 (15.1)	48 (13.7)	
age (mean (SD))	41.75 (12.61)	41.97 (12.64)	40.85 (12.49)	*0.136*
age groups, N%				
17-44 years old	987 (55.7)	781 (54.9)	206 (58.9)	*0.205*
45-70 years old	785 (44.3)	641 (45.1)	144 (41.1)	
gender, N%				
female	886 (50.0)	715 (50.3)	171 (48.9)	*0.676*
male	886 (50.0)	707 (49.7)	179 (51.1)	
region, N%				
urban	534 (30.1)	401 (28.2)	133 (38.0)	***<0.001***
rural	1238 (69.9)	1021 (71.8)	217 (62.0)	
marital status, N%				
unmarried 2	179 (10.1)	137 (9.6)	42 (12.0)	*0.224*
married	1593 (89.9)	1285 (90.4)	308 (88.0)	
educational level, N%				
primary and below	507 (28.6)	402 (28.3)	105 (30.0)	*0.811*
junior high school	787 (44.4)	634 (44.6)	153 (43.7)	
high school and above	478 (27.0)	386 (27.1)	92 (26.3)	
income [3], N%				
≥ ¥0, ≤ ¥5000	514 (29.5)	412 (29.4)	102 (30.0)	*0.665*
> ¥5000, ≤ ¥10000	533 (30.6)	432 (30.9)	101 (29.7)	
> ¥10000, ≤ ¥15000	268 (15.4)	209 (14.9)	59 (17.4)	
> ¥15000	425 (24.4)	347 (24.8)	78 (22.9)	
BMI (mean (SD))	25.14 (4.25)	25.23 (4.29)	24.76 (4.08)	*0.064*
BMI groups, N%				
< 24 kg/m2	750 (42.3)	594 (41.8)	156 (44.6)	*0.223*
≥ 24 kg/m2, < 28 kg/m2	627 (35.4)	499 (35.1)	128 (36.6)	
≥ 28 kg/m2	395 (22.3)	329 (23.1)	66 (18.9)	
smoking status, N%				
no	1296 (73.1)	1044 (73.4)	252 (72.0)	*0.639*
yes	476 (26.9)	378 (26.6)	98 (28.0)	

drinking status, N%				
no	1159 (65.4)	942 (66.2)	217 (62.0)	*0.152*
yes	613 (34.6)	480 (33.8)	133 (38.0)	
physical exercise, N%				
no	1236 (69.8)	993 (69.8)	243 (69.4)	*0.935*
yes	536 (30.2)	429 (30.2)	107 (30.6)	
dyslipidemia [4], N%				
no	1151 (65.0)	912 (64.2)	239 (68.3)	*0.168*
yes	620 (35.0)	509 (35.8)	111 (31.7)	
diabetes, N%				
no	1653 (93.3)	1322 (93.0)	331 (94.6)	*0.340*
yes	119 (6.7)	100 (7.0)	19 (5.4)	
center obesity, N%				
no	1052 (59.4)	849 (59.7)	203 (58.0)	*0.602*
yes	720 (40.6)	573 (40.3)	147 (42.0)	

The values were presented as mean (SD) for continuous variables or number (percentage) for categorical variables. Abbreviations: SD, standard deviation; SBP, systolic blood pressure; DBP, diastolic blood pressure; BMI, body mass index; [1] Obtained using the chi-squared test for categorical variables and 1-factor ANOVA for continuous variables. [2] Unmarried means single, divorced, separated, or widowed. [3] Income had 32 missing values. [4] Dyslipidemia had 1 missing value. Bold values are statistically significant.

Table S3 Relationship between low-sodium salt intake and SBP and DBP in different gender populations

Outcome	Low-sodium salt intake				*P for 1g increment*	*P for interaction*
	Q1	Q2	Q3	Per 1g increment		
SBP						*0.472*
Male						
Model 1	Ref	-1.263(-6.527, 4.000)	**-6.586(-11.631, -1.540)**	-0.636(-1.367, 0.096)	*0.088*	
Model 2	Ref	-2.387(-7.922, 3.147)	**-8.023(-13.451, -2.595)**	-0.759(-1.529, 0.011)	*0.053*	
Model 3	Ref	-2.184(-7.481, 3.114)	**-6.589(-11.822, -1.356)**	-0.489(-1.236, 0.258)	*0.198*	
Model 4	Ref	-1.460(-6.666, 3.747)	**-6.435(-11.708, -1.161)**	-0.444(-1.203, 0.314)	*0.249*	
Female						
Model 1	Ref	-4.360(-11.667, 2.947)	-5.151(-12.747, 2.444)	-0.742(-1.774, 0.291)	*0.158*	
Model 2	Ref	-4.982(-11.962, 1.999)	-6.339(-13.544, 0.865)	-0.748(-1.736, 0.240)	*0.137*	
Model 3	Ref	-3.430(-10.134, 3.273)	-3.841(-10.696, 3.013)	-0.585(-1.504, 0.335)	*0.211*	
Model 4	Ref	-4.585(-11.377, 2.208)	-4.565(-11.421, 2.290)	-0.700(-1.628, 0.227)	*0.138*	
DBP						*0.669*
Male						
Model 1	Ref	-0.987(-4.935, 2.961)	**-4.306(-8.091, -0.521)**	-0.483(-1.029, 0.062)	*0.082*	
Model 2	Ref	-1.655(-5.780, 2.471)	**-5.228(-9.274, -1.182)**	**-0.571(-1.141, -0.001)**	***0.050***	
Model 3	Ref	-1.668(-5.524, 2.188)	**-3.924(-7.733, -0.115)**	-0.317(-0.858, 0.223)	*0.248*	
Model 4	Ref	-0.986(-4.745, 2.773)	-3.453(-7.261, 0.354)	-0.239(-0.782, 0.305)	*0.387*	
Female						
Model 1	Ref	-4.095(-8.717, 0.528)	-3.057(-7.862, 1.748)	-0.391(-1.047, 0.266)	*0.242*	
Model 2	Ref	**-4.792(-9.313, -0.270)**	-3.591(-8.258, 1.076)	-0.385(-1.029, 0.259)	*0.240*	
Model 3	Ref	-3.616(-7.605, 0.373)	-1.223(-5.302, 2.856)	-0.236(-0.788, 0.316)	*0.399*	
Model 4	Ref	**-4.082(-8.141, -0.022)**	-1.595(-5.693, 2.502)	-0.301(-0.859, 0.258)	*0.290*	

Model 1: Not adjusted. Model 2: adjusted for age, region, marital status, education level, and income. Model 3: adjusted for model 2 + smoking status, drinking status, physical exercise, and BMI. Model 4: adjusted for model 3 + dyslipidemia, diabetes and central obesity. Bold values are statistically significant.

Table S4 Relationship between low-sodium salt intake and SBP and DBP in different age groups

Outcome	Low-sodium salt intake				*P for 1g increment*	*P for interaction*
	Q1	Q2	Q3	Per 1g increment		
SBP						*0.753*
17-44 years old						
Model 1	Ref	-1.215(-6.118, 3.688)	-2.077(-6.807, 2.652)	-0.239(-0.892, 0.415)	*0.473*	
Model 2	Ref	-3.059(-7.709, 1.591)	**-4.672(-9.203, -0.140)**	-0.466(-1.084, 0.152)	*0.139*	
Model 3	Ref	-2.124(-6.367, 2.118)	-3.563(-7.676, 0.550)	-0.339(-0.898, 0.221)	*0.234*	
Model 4	Ref	-2.414(-6.665, 1.837)	-3.511(-7.585, 0.564)	-0.358(-0.912, 0.197)	*0.205*	
45-70 years old						
Model 1	Ref	-3.586(-11.905, 4.732)	-7.819(-16.313, 0.675)	-0.818(-2.054, 0.418)	*0.193*	
Model 2	Ref	-3.932(-12.631, 4.767)	**-9.125(-18.201, -0.048)**	-0.925(-2.239, 0.390)	*0.166*	
Model 3	Ref	-3.627(-12.003, 4.749)	-6.079(-15.052, 2.894)	-0.438(-1.732, 0.856)	*0.504*	
Model 4	Ref	-3.416(-11.737, 4.905)	-5.488(-14.510, 3.535)	-0.278(-1.569, 1.012)	*0.670*	
DBP						*0.433*
17-44 years old						
Model 1	Ref	-0.748(-4.449, 2.953)	-0.993(-4.563, 2.577)	-0.091(-0.585, 0.402)	*0.715*	
Model 2	Ref	-1.891(-5.708, 1.926)	-2.260(-5.979, 1.459)	-0.191(-0.697, 0.315)	*0.458*	
Model 3	Ref	-1.077(-4.329, 2.176)	-1.133(-4.287, 2.020)	-0.064(-0.491, 0.364)	*0.770*	
Model 4	Ref	-1.373(-4.609, 1.863)	-1.122(-4.224, 1.979)	-0.086(-0.507, 0.336)	*0.689*	
45-70 years old						
Model 1	Ref	-4.677(-9.581, 0.227)	**-6.619(-11.627, -1.612)**	**-0.753(-1.485, -0.020)**	***0.044***	
Model 2	Ref	-4.743(-9.913, 0.427)	**-7.306(-12.700, -1.913)**	**-0.811(-1.595, -0.026)**	***0.043***	
Model 3	Ref	-4.789(-9.729, 0.150)	-4.887(-10.179, 0.405)	-0.452(-1.221, 0.317)	*0.247*	
Model 4	Ref	-4.623(-9.593, 0.347)	-4.598(-9.987, 0.791)	-0.378(-1.155, 0.398)	*0.337*	

Model 1: Not adjusted. Model 2: adjusted for gender, region, marital status, education level, and income. Model 3: adjusted for model 2 + smoking status, drinking status, physical exercise, and BMI. Model 4: adjusted for model 3 + dyslipidemia, diabetes and central obesity. Bold values are statistically significant.

Table S5 Relationship between low-sodium salt intake and SBP and DBP in different region people

Outcome	Low-sodium salt intake				*P for 1g increment*	*P for interaction*
	Q1	Q2	Q3	Per 1g increment		
SBP						*0.795*
Urban						
Model 1	Ref	-1.714(-7.930, 4.503)	-2.353(-8.731, 4.025)	-0.573(-1.382, 0.236)	*0.164*	
Model 2	Ref	-4.459(-10.576, 1.658)	-3.060(-9.343, 3.224)	-0.656(-1.422, 0.110)	*0.092*	
Model 3	Ref	-4.265(-9.972, 1.442)	-1.273(-7.141, 4.596)	-0.459(-1.174, 0.257)	*0.207*	
Model 4	Ref	-4.472(-10.202, 1.257)	-1.702(-7.606, 4.202)	-0.462(-1.179, 0.256)	*0.205*	
Rural						
Model 1	Ref	-2.318(-8.782, 4.145)	-6.134(-12.397, 0.129)	-0.485(-1.429, 0.458)	*0.312*	
Model 2	Ref	-2.812(-9.206, 3.583)	**-8.616(-14.825, -2.406)**	-0.818(-1.757, 0.121)	*0.087*	
Model 3	Ref	-2.285(-8.333, 3.762)	**-7.558(-13.526, -1.591)**	-0.637(-1.533, 0.260)	*0.163*	
Model 4	Ref	-2.624(-8.607, 3.360)	**-6.950(-12.808, -1.092)**	-0.532(-1.411, 0.346)	*0.234*	
DBP						*0.364*
Urban						
Model 1	Ref	-2.787(-7.523, 1.950)	-3.544(-8.404, 1.316)	-0.604(-1.220, 0.013)	*0.055*	
Model 2	Ref	-4.267(-9.149, 0.616)	-3.298(-8.313, 1.716)	**-0.621(-1.232, -0.010)**	***0.046***	
Model 3	Ref	**-4.364(-8.718, -0.011)**	-2.053(-6.529, 2.424)	-0.486(-1.032, 0.060)	*0.081*	
Model 4	Ref	**-4.477(-8.873, -0.081)**	-2.265(-6.796, 2.265)	-0.486(-1.037, 0.065)	*0.083*	
Rural						
Model 1	Ref	-1.981(-5.984, 2.021)	-3.442(-7.321, 0.436)	-0.201(-0.785, 0.384)	*0.499*	
Model 2	Ref	-2.370(-6.443, 1.702)	**-4.729(-8.684, -0.775)**	-0.344(-0.941, 0.253)	*0.257*	
Model 3	Ref	-1.873(-5.593, 1.847)	-3.132(-6.803, 0.539)	-0.108(-0.657, 0.441)	*0.698*	
Model 4	Ref	-1.931(-5.581, 1.718)	-2.660(-6.233, 0.913)	-0.038(-0.571, 0.495)	*0.888*	

Model 1: Not adjusted. Model 2: adjusted for age, gender, marital status, education level, and income. Model 3: adjusted for model 2 + smoking status, drinking status, physical exercise, and BMI. Model 4: adjusted for model 3 + dyslipidemia, diabetes and central obesity. Bold values are statistically significant.

Table S6 Relationship between low-sodium salt intake and SBP and DBP

Outcome	Low-sodium salt intake				*P for 1g increment*
	Q1	Q2	Q3	Per 1g increment	
SBP	Ref	-3.105(-7.283, 1.073)	**-4.961(-9.142, -0.779)**	-0.452(-1.036, 0.132)	*0.129*
DBP	Ref	**-2.845(-5.559, -0.131)**	-2.696(-5.412, 0.020)	-0.235(-0.615, 0.145)	*0.224*

Adjusting for age, gender, region, marital status, education level, income, smoking status, drinking status, physical exercise, BMI, dyslipidemia, diabetes, central obesity and other salt intake. Bold values are statistically significant.

Table S7 Relationship between low-sodium salt intake and SBP and DBP in different subgroups

Outcome	Low-sodium salt intake				*P for 1g increment*	*P for interaction*
	Q1	Q2	Q3	Per 1g increment		
SBP [1]						*0.475*
Male	Ref	-1.596(-6.824, 3.633)	**-6.312(-11.605, -1.019)**	-0.412(-1.175, 0.351)	*0.287*	
Female	Ref	-4.703(-11.526, 2.121)	-4.926(-11.931, 2.078)	-0.764(-1.716, 0.188)	*0.115*	
DBP [1]						*0.669*
Male	Ref	-0.874(-4.646, 2.899)	-3.555(-7.374, 0.264)	-0.258(-0.805, 0.289)	*0.353*	
Female	Ref	-4.037(-8.117, 0.044)	-1.458(-5.647, 2.731)	-0.287(-0.861, 0.287)	*0.325*	
SBP [2]						*0.743*
17-44 years old	Ref	-2.417(-6.680, 1.846)	-3.550(-7.685, 0.585)	-0.368(-0.938, 0.201)	*0.204*	
45-70 years old	Ref	-3.096(-11.402, 5.210)	-5.134(-14.141, 3.873)	-0.290(-1.575, 0.995)	*0.656*	
DBP [2]						*0.442*
17-44 years old	Ref	-1.385(-4.625, 1.854)	-1.315(-4.457, 1.827)	-0.124(-0.556, 0.307)	*0.570*	
45-70 years old	Ref	-4.443(-9.408, 0.522)	-4.399(-9.783, 0.986)	-0.385(-1.158, 0.389)	*0.327*	
SBP [3]						*0.778*
Urban	Ref	-4.481(-10.243, 1.280)	-1.772(-8.038, 4.494)	-0.555(-1.335, 0.226)	*0.162*	
Rural	Ref	-2.625(-8.629, 3.380)	**-6.951(-12.837, -1.065)**	-0.531(-1.413, 0.352)	*0.237*	
DBP [3]						*0.357*
Urban	Ref	**-4.463(-8.884, -0.043)**	-2.160(-6.967, 2.648)	-0.545(-1.144, 0.055)	*0.075*	
Rural	Ref	-1.946(-5.608, 1.715)	-2.683(-6.273, 0.907)	-0.039(-0.575, 0.496)	*0.884*	

[1] Adjusting for age, region, marital status, education level, income, smoking status, drinking status, physical exercise, BMI, dyslipidemia, diabetes, central obesity and other salt intake; [2] Adjusting for gender, region, marital status, education level, income, smoking status, drinking status, physical exercise, BMI, dyslipidemia, diabetes, central obesity and other salt intake; [3] Adjusting for age, gender, marital status, education level, income, smoking status, drinking status, physical exercise, BMI, dyslipidemia, diabetes, central obesity and other salt intake. Bold values are statistically significant.
